# Supplementary material for: Digital tools for delivery of dementia education for caregivers of persons with dementia: A systematic review and meta-analysis of impact on caregiver distress and depressive symptoms
Source: PLoS One. 2023 May 17;18(5):e0283600. doi: 10.1371/journal.pone.0283600 (PMC10191337; doi:10.1371/journal.pone.0283600)
Supplement: S1 Appendix — (PDF) [file pone.0283600.s002.pdf]

S1 Appendix. Search syntax.

PsycINFO MeSH terms and keywords logic grid

physicians OR general practitioners OR health personnel OR nurses OR nursing OR medical students OR health care services OR primary health care OR health care delivery OR medical personnel OR allied health personnel OR paraprofessional personnel OR physical therapists OR home care personnel OR mental health personnel OR occupational therapists OR recreation therapy OR professional personnel OR nursing students OR (healthcare team OR doctor OR specialist OR care assistant).mp	dementia OR Alzheimer's disease OR Parkinson's disease OR vascular dementia OR dementia with Lewy bodies OR semantic dementia OR neurodegenerative diseases OR cognitive impairment OR neurodegeneration OR (frontotemporal dementia OR early onset dementia OR young onset dementia OR late onset dementia OR mild cognitive impairment OR late elderly dementia).mp	online education OR computer assisted instruction OR distance education OR internet OR computer applications OR computer searching OR computer training OR technology OR machine learning OR virtual classrooms OR multimedia OR websites OR electronic learning OR mobile devices OR telemedicine OR teleconferencing OR videotape instruction OR telecommunications media OR telephone systems OR (online learning OR online training OR online program OR online tool OR online resources OR computer-based OR computerized OR asynchronous OR portal OR learning application OR videoconferencing OR telephone-based OR telephone-delivered).mp
