# Supplementary material for: Digital tools for delivery of dementia education for caregivers of persons with dementia: A systematic review and meta-analysis of impact on caregiver distress and depressive symptoms
Source: PLoS One. 2023 May 17;18(5):e0283600. doi: 10.1371/journal.pone.0283600 (PMC10191337; doi:10.1371/journal.pone.0283600)
Supplement: S1 Table — (PDF) [file pone.0283600.s003.pdf]

S1 Table. Adapted quality rating criteria for randomized controlled trial studies (studies not included in meta-analysis)

Quality criteria	Specific questions relating to rating considerations	Rating
1. Did the research question clearly address a focused issue?	• Was the research question focused on the target population, and the intervention?<br>• Were outcomes in research considered?	0 = no; 1 = partially; 2 = yes
2. Was the assignment of participants randomized to the intervention or the control group?	• How was randomization achieved?<br>• Was randomization concealed from researcher or participants?	0 = no; 1 = partially; 2 = yes
3. Were all of the participants properly accounted for at the conclusion of the intervention?	• Were the participants analyzed in groups to which they were randomized?	0 = no; 1 = partially; 2 = yes
4. Were participants, and care recipients blind to whether they were in the intervention or the control group?	• Consider reporting of participant blinding to intervention/control, and blinding of researcher(s) to condition in assessment of outcomes.	0 = no; 1 = partially; 2 = yes
5. Were the intervention and control groups similar at the start of the study?	• Were the intervention and control groups similar in terms of their age, sex, social class...etc?	0 = no; 1 = partially; 2 = yes
6. Aside from the intervention group, where the intervention and control groups treated equally?	• Here also consider duration of intervention or placebo.	0 = no; 1 = partially; 2 = yes
7. How large was the treatment effect?	• Was/were the primary outcome(s) clearly stated?<br>• What were the results for each outcome?	0 = no; 1 = partially; 2 = yes
8. How precise was the estimate of the treatment effect?	• What were the confidence limits?	0 = no; 1 = partially; 2 = yes

9. Can the results be applied to the local population?	• How similar were the participants to the population to which the recommendations were applied?	0 = no; 1 = partially; 2 = yes
10. Were all clinically important outcomes considered?	• Was there other information about the outcomes that was missed?	0 = no; 1 = partially; 2 = yes
11. Are benefits worth the harms and costs?	• Here consider possibility of harm in the control condition, if education piece was missed.	0 = no; 1 = partially; 2 = yes<br>Range: 0-21

Note: 1-7 = low range, 7-15 = medium range, 16-21 = high range
