# Supplementary material for: Digital tools for delivery of dementia education for caregivers of persons with dementia: A systematic review and meta-analysis of impact on caregiver distress and depressive symptoms
Source: PLoS One. 2023 May 17;18(5):e0283600. doi: 10.1371/journal.pone.0283600 (PMC10191337; doi:10.1371/journal.pone.0283600)
Supplement: S2 Table — (PDF) [file pone.0283600.s004.pdf]

S2 Table. Adapted quality rating criteria for non-randomized controlled trial studies (studies not included in meta-analysis)

Quality criteria	Specific questions relating to rating considerations	Rating
1. Are the research aims and questions/hypotheses clearly stated?	• Do(es) the author(s) clearly state what they plan to research?	0 = no; 1 = partially; 2 = yes
2. Are ethical issues addressed?	• Do(es) the author(s) state that ethical approval was sought?	0 = no; 1 = partially; 2 = yes
3. Are the methodology/study design appropriate to the research question and rationale for choice evident?	• Do(es) the author state what research methodology they have chosen?<br>• Is the chosen methodology appropriate to research question?<br>• Does the author(s) justify the research design used?	0 = no; 1 = partially but with weaknesses/missing info; 2 = yes
4. Are the sample size, selection and description appropriate?	• Do(es) the autho(s) clearly state how the study sample size was identified?<br>• Do(es) the sample size appear to be large enough?<br>• Do(es) the author adequately describe the sample (e.g., gender, age, relationship to care receiver) so that the reader can determine transferability of findings?	0 = no; 1 = partially but with weaknesses/missing info; 2 = yes
5. Are the method(s) of data collection appropriate, reliable, and valid?	• Do(es) the author(s) justify that the measure is suitable for this population?<br>• Do(es) the author(s) use measures that measure the desired constructs?<br>• Do(es) the author(s) indicate that the measures have good psychometric properties?<br>• Do(es) the author(s) indicate that the measures used have demonstrated validity?	0 = no; 1 = partially but with weaknesses/missing info; 2 = yes
6. Are the method(s) of data analysis reliable and valid?	• Do(e)s the author(s) state which statistic tests were used?<br>• Do(es) the author(s) use statistical tests that appear to be appropriate to the nature of the data collected?<br>• Were statistical tests appropriate to research question?<br>• Do(es) the author(s) provide evidence of statistical findings or state levels of significance?	0 = no; 1 = partially but with weaknesses/missing info; 2 = yes
7. Are the findings and discussion clearly stated and appropriate?	• Do(es) the author(s) present the statistical data in a clear manner, or clearly differentiate between significant or non-significant findings?	0 = no; 1 = partially; 2 = yes

8. Can the results be applied to the local population?	• How similar are the participants to the population to which the recommendations are applied?	0 = no; 1 = partially; 2 = yes
9. Were all clinically important outcomes considered?	• Was there other information about the outcomes which was missed?	0 = no; 1 = partially; 2 = yes<br><br>Range: 0-18

Note: 1-6 = low range, 7-12 = medium range, 13-18 = high range
