# Supplementary material for: Digital tools for delivery of dementia education for caregivers of persons with dementia: A systematic review and meta-analysis of impact on caregiver distress and depressive symptoms
Source: PLoS One. 2023 May 17;18(5):e0283600. doi: 10.1371/journal.pone.0283600 (PMC10191337; doi:10.1371/journal.pone.0283600)
Supplement: S3 Table — (PDF) [file pone.0283600.s005.pdf]

S5 Table. Characteristics of included studies.

Description	*N* (%)
Country of study	
United States	22 (78)
Netherlands	3 (10)
Poland/Spain/Denmark	1 (4)
France	1 (4)
China	1 (4)
Caregivers	
Spouse/Partner/Significant Other	15 (29)
Adult Child	11 (23)
Caregiver of Veteran	2 (4)
Sibling	2 (4)
Other relative	14 (27)
Volunteer	1 (2)
Professional Caregiver	1 (2)
Caregiver (relationship unknown)	5 (9)
Location	
Urban	3 (10)
Rural	1 (4)
Mixed	1 (4)
Unknown	23 (82)
CASP Study Quality NIMA-RCT	
High	6 (100)
Medium	0 (Hodges et al.)
Low	0 (Hodges et al.)
CASP Study Quality NIMA-non-RCT	
High	2 (25)
Medium	6 (75)
Low	0 (Hodges et al.)

Note: CASP = Critical Appraisal Skills Programme, NIMA = Not included in meta-analysis - CASP Study Quality was performed only for studies not included in the meta-analysis; number of health professionals is greater, since some of the studies used multiple types of health professionals in their interventions.
