# Supplementary material for: Digital tools for delivery of dementia education for caregivers of persons with dementia: A systematic review and meta-analysis of impact on caregiver distress and depressive symptoms
Source: PLoS One. 2023 May 17;18(5):e0283600. doi: 10.1371/journal.pone.0283600 (PMC10191337; doi:10.1371/journal.pone.0283600)
Supplement: S4 Table — (PDF) [file pone.0283600.s006.pdf]

S6 Table. Risk of bias assessment for RCT studies not included in meta-analysis.

Author, Year	Random sequence generation	Allocation concealment	Blinding of participants	Blinding of outcome assessment	Incomplete outcome data	Selective reporting
Czaja, 2018	?	?	?	?	+	?
Goodman, 1990	?	?	+	?	+	-
Goodman & Pynoos, 1990	?	?	?	?	-	-
Hattink, 2015	?	?	?	?	-	-
Hicken, 2017	?	?	?	?	?	?
Mavandadi, Wray, et al., 2017	+	?	+	?	+	-
Mavandadi, Wright, et al., 2017	-	-	?	?	-	+
Wijma, 2018	?	?	?	?	-	+

Note: RCT = Randomized Controlled Trial, ? =unknown risk of bias, - = low risk bias, + = high risk bias
