# Supplementary material for: Digital tools for delivery of dementia education for caregivers of persons with dementia: A systematic review and meta-analysis of impact on caregiver distress and depressive symptoms
Source: PLoS One. 2023 May 17;18(5):e0283600. doi: 10.1371/journal.pone.0283600 (PMC10191337; doi:10.1371/journal.pone.0283600)
Supplement: S5 Table — (PDF) [file pone.0283600.s007.pdf]

S7 Table. Risk of bias assessment for RCT studies included in meta-analysis.

Author, Year	Random sequence generation	Allocation concealment	Blinding of participants	Blinding of outcome assessment	Incomplete outcome data	Selective reporting
Beauchamp, 2005	?	-	?	?	-	-
Czaja, 2013	?	?	-	?	-	-
Gallagher-Thompson, 2010	-	-	+	?	+	-
Glueckauf, 2007	?	?	?	?	+	-
Kajiyama, 2013	?	?	?	?	-	-
Martindale-Adams, 2013	?	?	?	?	-	-
Núñez-Naveira, 2016	-	?	?	?	-	-
Cristancho-LaCroix, 2015	-	?	?	?	-	-
Finkel, 2007	?	?	-	-	+	+
Gant, 2007	?	?	?	?	-	-
Steffen, 2000	?	?	?	?	-	-
Steffen, 2016	-	-	-	?	-	-
Kwok, 2013	-	?	?	?	+	-
Boots, 2018	+	+	-	?	+	-

Note: RCT = Randomized Controlled Trial, ? =unknown risk bias, - = low risk bias, + = high risk bias
