# Supplementary material for: Digital tools for delivery of dementia education for caregivers of persons with dementia: A systematic review and meta-analysis of impact on caregiver distress and depressive symptoms
Source: PLoS One. 2023 May 17;18(5):e0283600. doi: 10.1371/journal.pone.0283600 (PMC10191337; doi:10.1371/journal.pone.0283600)
Supplement: S6 Table — (PDF) [file pone.0283600.s008.pdf]

S8 Table. Quality of studies included in meta-analysis conveyed with Grading of Recommendations, Assessment, Development and Evaluations (Pourahmadi et al.).

Outcomes	No. of participants (studies)	Certainty of the evidence (Pourahmadi et al.)
Depression assessed with: Center for Epidemiological Studies Depression Scale (CES-D)	868 (8 RCTs)	⊕⊕◯◯ LOW
Observable Behavioral Problems assessed with: Revised Memory and Behavior Problem Checklist (RMBPC)	592 (8 RCTs)	⊕◯◯◯ VERY LOW
Caregiving Self-Efficacy assessed with: Caregiving Self-Efficacy Scale (CSES)	292 (7 RCTs)	⊕⊕◯◯ LOW
Caregiver Burden assessed with: Zarit Burden Interview (ZBI)	241 (3 RCTs)	⊕⊕◯◯ LOW
Depression assessed with: Beck Depression Inventory (BDI-II)	134 (3 RCTs)	⊕◯◯◯ VERY LOW
Positive Caregiving Experience assessed with: Positive Aspects of Caregiving (PAC)	444 (3 RCTs)	⊕⊕◯◯ LOW
