# Supplementary material for: Digital tools for delivery of dementia education for caregivers of persons with dementia: A systematic review and meta-analysis of impact on caregiver distress and depressive symptoms
Source: PLoS One. 2023 May 17;18(5):e0283600. doi: 10.1371/journal.pone.0283600 (PMC10191337; doi:10.1371/journal.pone.0283600)
Supplement: S7 Table — (PDF) [file pone.0283600.s009.pdf]

**S9 Table.** Teaching and learning approaches.

| Teaching/learning approaches | *N* (%) |
|---|---|
| Online-based learning | |
| Information/educational modules | 8 (12) |
| Website platform | 1 (2) |
| Live interactive classes with instructor | 2 (3) |
| Telephone-based learning | |
| Telephone support group | 3 (4) |
| Telephone educational sessions | 8 (12) |
| Teleconferences with instructor | 1 (2) |
| Home telehealth device | 1 (2) |
| Telephone-accessed taped lectures | 2 (3) |
| Videophone support group sessions | 1 (2) |
| Computer-telephone integration system | 1 (2) |
| Mobile device/Smartphone/tablet | 1 (2) |
| Video-based learning | |
| Video-instructional/visual resources | 13 (21) |
| DVD- role playing and narration | 2 (3) |
| Video-role playing | 1 (2) |
| Educational Webnovella videos | 1 (2) |
| Virtual Reality Simulation | 1 (2) |
| Other resources | |
| Textual resources | 8 (12) |
| Interactive feedback | 2 (3) |
| Interactive exercises | 1 (2) |
| Knowledge tests | 2 (3) |
| References to other resource websites | 3 (4) |

*Note:* Number of studies is greater, since some of the studies used multiple approaches to teaching or learning.
