# Supplementary material for: Digital tools for delivery of dementia education for caregivers of persons with dementia: A systematic review and meta-analysis of impact on caregiver distress and depressive symptoms
Source: PLoS One. 2023 May 17;18(5):e0283600. doi: 10.1371/journal.pone.0283600 (PMC10191337; doi:10.1371/journal.pone.0283600)
Supplement: Attachment — Submitted filename: Response to reviewers.docx [file pone.0283600.s010.docx]

Lisa Susan Wieland
Academic Editor
PLOS ONE

Thank you for submitting your manuscript to PLOS ONE. After careful consideration, we feel that it has merit but does not fully meet PLOS ONE’s publication criteria as it currently stands. Therefore, we invite you to submit a revised version of the manuscript that addresses the points raised during the review process.

Thank you for providing us with this opportunity to revise the manuscript.

In addition, please address the following:

1) PRISMA is a systematic review reporting recommendation, not a recommendation for systematic review conduct

Thank you for pointing out our sloppy use of language – we have addressed this error in the revised manuscript.

2) there is not a standard Cochrane data extraction form. Please clarify the source of this form or omit the mention

Thank you we have deleted the reference to a standard form.

3) Cochrane risk of bias is conducted for each domain of study conduct. How did the authors evaluate overall risk of bias for the studies?

We have added the citation to the Cochrane training handbook. Higgins JPT, Savović J, Page MJ, Elbers RG, Sterne JAC. Chapter 8: Assessing risk of bias in a randomized trial. In: Higgins JPT, Thomas J, Chandler J, Cumpston M, Li T, Page MJ, Welch VA (editors). Cochrane Handbook for Systematic Reviews of Interventions version 6.2 (updated February 2021). Cochrane, 2021. Available from [www.training.cochrane.org/handbook](http://www.training.cochrane.org/handbook). We also specified that when we reported on the risk of bias for the result from each study this was based on the least favourable assessment across the domains considered as per Higgins et al., 2021 on page 7 of the revised manuscript.

4) GRADE is not used for the quality of studies but rather the quality (or confidence) of effect estimates. Please correct the use of GRADE in this paper.

Thank you for pointing out our sloppy use of language – we have addressed this error in the revised manuscript.

5) Funnel plots for assessing the possibility of publication bias are not appropriate with fewer than at least 10 studies.

Thank you for pointing out our error and we have deleted the two funnel plots.

Reviewers' comments:

Reviewer's Responses to Questions

**Comments to the Author**

1. Is the manuscript technically sound, and do the data support the conclusions?

The manuscript must describe a technically sound piece of scientific research with data that supports the conclusions. Experiments must have been conducted rigorously, with appropriate controls, replication, and sample sizes. The conclusions must be drawn appropriately based on the data presented.

Reviewer #1: Yes

Reviewer #2: No

2. Has the statistical analysis been performed appropriately and rigorously?

Reviewer #1: Yes

Reviewer #2: Yes

3. Have the authors made all data underlying the findings in their manuscript fully available?

The [PLOS Data policy](http://www.plosone.org/static/policies.action#sharing) requires authors to make all data underlying the findings described in their manuscript fully available without restriction, with rare exception (please refer to the Data Availability Statement in the manuscript PDF file). The data should be provided as part of the manuscript or its supporting information, or deposited to a public repository. For example, in addition to summary statistics, the data points behind means, medians and variance measures should be available. If there are restrictions on publicly sharing data—e.g. participant privacy or use of data from a third party—those must be specified.

Reviewer #1: Yes

Reviewer #2: Yes

4. Is the manuscript presented in an intelligible fashion and written in standard English?

PLOS ONE does not copyedit accepted manuscripts, so the language in submitted articles must be clear, correct, and unambiguous. Any typographical or grammatical errors should be corrected at revision, so please note any specific errors here.

Reviewer #1: Yes

Reviewer #2: Yes

5. Review Comments to the Author

Please use the space provided to explain your answers to the questions above. You may also include additional comments for the author, including concerns about dual publication, research ethics, or publication ethics. (Please upload your review as an attachment if it exceeds 20,000 characters)

Reviewer #1: A well-structured and thorough review, of clinical and social importance. Your focus on digital technologies seems particularly apt in current COVID-19 circumstances, and very applicable to the current environment of healthcare delivery. Some structural changes and better situation of your discussion within the current research would improve the applicability of this review outside of your own working context.

• I would like to see justification of why only English-language studies were included, and see this addressed under limitations.

We have included the reasoning regarding our justification in the paper e.g., limitations – inability to speak or understand other languages by the reviewers, also limitations of available university search engine, thus publications in English only (pg. 8-9).

• A critical and structured reflection on your review of the strengths and limitations of your study should be included at the beginning of the discussion.

We agree and we have made this change in the revised manuscript – please see the first paragraph of the revised discussion on p. 17 and 18.

• In general, I think your discussion and clinical implications could have a wider scope. Though it’s great that you spoke with a consultant about this work, I don’t think it should be the main basis of this section of your discussion.

We have revised the discussion and hopefully have sufficiently broadened the scope. We focused on limitations in the review and gaps identified to expand on future directions that could have clinical implications on pages 20-23.

• I would like to see your discussion better situated within current research and clinical practice outside of this one clinical setting to improve the scope and impact of this review.

We agree, and have linked the findings to the current research and to recent reviews in the similar area (see discussion pages 18-20. We discussed the clinical implications in the prior point, but refer you to pages 20-23 of the revised discussion.

• Your findings around gendered caregiving are really interesting, and I think you can make more of these in the discussion and recommendations for future research and policy.

Thank you we agree, and we have tried to incorporate gendered caregiving and sex issues more into our discussion on pages 21-23.

• You need a proper conclusion that brings together all elements of the paper, outside of the views of authors and one consultant.

We agree, and we have added a conclusion section in the revised manuscript on pages 23-24.

Reviewer #2: Studying digital delivery of interventions for carers is very timely given the global pandemic. As the authors state, there is benefit to carers being able to access remote and asynchronous interventions. The authors also raise an excellent point that research points to male-female differences in coping with caring, such that we might expect different responses to interventions depending on coping style. Unfortunately, however, the paper tries to cover too much ground and does not quite cover any of it satisfactorily given the mismatch between aims and reported findings. There are very many reviews of non-pharmacological interventions for family carers of people with dementia and a review now needs to be excellent quality if it is to be worth publishing. The following major revisions would be required if this review is to usefully add to the literature:

1) Awareness of relevant recent reviews

The authors should update their background literature to include citations relevant recent reviews, especially those that include a technology component e.g.
Frias, C. E., Garcia‐Pascual, M., Montoro, M., Ribas, N., Risco, E., & Zabalegui, A. (2020). Effectiveness of a psychoeducational intervention for caregivers of people with dementia with regard to burden, anxiety and depression: a systematic review. Journal of advanced nursing, 76(3), 787-802.

We have made more of an effort to include existing and relevant reviews with a technology-based component, including the recommended one above. Thank you for noting this. This is briefly discussed in the introduction, but is a focus in the discussion on pages 19-20.

2) Clarity of aim(s) &associated presentation of mateerial
The stated aims of this study would be appropriate for a scoping review (what tech available, how is it evaluated, which outcomes?). However, for a meta-analysis, there should be a clear question that would best be answered through use of meta-analysis. If the authors wish to focus on whether results are reported separately according to gender or sex this should be part of the aim; conversely, if there is to be a ‘focus on studies with pre-post measures’ (p. 7) or ‘focus on motivation and accessibility’ (p. 7), then findings relating to these issues should be provided. The authors should re-write the paper so that the aims and content are consistent with each other.

Although the questions asked were broad and seem more characteristic of a scoping review, we wanted use these to extract the relevant information pertaining to our singular question; what is the existing evidence for digitally-based dementia interventions for caregivers with pre-post measures. In effort to clarify this, we changed the writing to reflect our question. As noted, systematic reviews usually are written very precisely to reflect specific key elements of a review question; for intervention questions, these are the population, intervention, comparison, and outcome. We have attempted to clarify this more in the review. The intended aim of the present study was to carry out a systematic review and describe the data qualitatively. The meta-analysis was not planned originally, but with the consistency in used measures and outcomes in some of the studies, we decided to carry out the quantitative synthesis or the meta-analysis on an ad hoc basis. We clarified this more in the study, and appropriately changed the wording and the intent. Also, the separate reporting according to sex and gender was not a part of our original question, this is something that came out of the review, and thus we decided to discuss it ad hoc, as it seemed pertinent to modern caregivers, as well as the differences in gender, health and care. We have clarified this in the study as well on page 9.

3) Appropriateness of methods
3.1 Please ensure that there is a match between the study selection (based on eligibility criteria) and the proposed synthesis strategy (in this case, meta-analysis). Single group pre-post studies are not relevant to a meta-analysis of RCTs

A meta-analysis was not the intent nor focus of this review, it was performed ad hoc. The intent of this study was a systematic review of existing modes of remote to be described qualitatively thus we focused on digital education on dementia for caregivers with a single group pre-post studies. We have made an effort to clarify this more in the paper (pg. 9).

3.2 Please include search dates & explain why there were two searches with two separate PRISMA flow-charts.

The two PRISMA flow charts correspond to the initial search which was performed in the previous year, the second PRISMA chart corresponds to the updated review which would capture any new studies within the year since the initial search. This reasoning is now included in the paper (pg. 9, 40, 41).

4) Results
4.1 At present, there is a good deal of space taken up reporting excluded studies. N=28 studies were deemed eligible of which 14 were included in the meta-analysis.

The excluded studies were also the focus of the review (contain existing modes of remote, digital education on dementia for caregivers with a single group pre-post studies thus the findings are deemed pertinent), and their data is accordingly qualitatively described.

4.2 Reporting of results should be checked for accuracy. Specifically:
- the self-efficacy analysis indicates that control is favoured over intervention with an effect size of .21 (CI -0.03, 0.44) rater than the reported ‘no effect’. Please check and revise paper accordingly.

We have made the corrections as noted.

- the PAC analysis indicates no effect, but this is reported as ‘positive effect on PAC’ (p. 17, p. 19). Please check and revise paper accordingly.

Thank you, we have made the suggested correction.

- Table 8 – number of participants differs between forest plots and table 8 for CES-D (depression) and self-efficacy

Thank you for noting this, we have made the correction and the forest plots are deleted.

4.3 Missing – summary of ‘control’ conditions

We have added these summaries for each control condition that were missing from each study in Table 3.

5) Discussion
- Please compare findings to those of other relevant reviews & consider why there may be discrepancies

Thank you for your recommendation, we have included a section comparing our findings to other relevant reviews and why there might be discrepancies on pages 18-21.

- Please add a ‘limitations’ section with appropriate critical appraisal of the methodology used e.g. inclusion of ‘live’ phone interventions when stated interest in ‘asynchronous’ interventions

We did not wish to convey a sole interest in asynchronous interventions, merely digitally based interventions and have revised the abstract and study aims to make this more clear.

- p. 21 to 23 appears to be a report of a discussion with a consultant on the impact of COVID-19 on carers. I’m assuming that this paper has been submitted elsewhere previously & it was suggested that ‘clinical implications’ were considered? It would be helpful if the findings from this useful chat were distilled to the points most pertinent to the review.

This was not an original consideration in the review, but was added to help tie the findings to existing clinical implications, and better situate our findings in context of real care. As recommended, we have decided to exclude this section as it did not seem pertinent to the review. We discuss the limitations of the review on page 18 and again on pages 20-23.

6. PLOS authors have the option to publish the peer review history of their article ([what does this mean?](https://journals.plos.org/plosone/s/editorial-and-peer-review-process#loc-peer-review-history)). If published, this will include your full peer review and any attached files.
