# Supplementary material for: Digital tools for delivery of dementia education for caregivers of persons with dementia: A systematic review and meta-analysis of impact on caregiver distress and depressive symptoms
Source: PLoS One. 2023 May 17;18(5):e0283600. doi: 10.1371/journal.pone.0283600 (PMC10191337; doi:10.1371/journal.pone.0283600)
Supplement: Attachment — Submitted filename: Response to Reviews.docx [file pone.0283600.s011.docx]

Reviewers' comments:

Reviewer's Responses to Questions

**Comments to the Author**

*1. If the authors have adequately addressed your comments raised in a previous round of review and you feel that this manuscript is now acceptable for publication, you may indicate that here to bypass the “Comments to the Author” section, enter your conflict of interest statement in the “Confidential to Editor” section, and submit your "Accept" recommendation.*

*Reviewer #3: All comments have been addressed*

 We thank the reviewer for noting this.

*2. Is the manuscript technically sound, and do the data support the conclusions?

The manuscript must describe a technically sound piece of scientific research with data that supports the conclusions. Experiments must have been conducted rigorously, with appropriate controls, replication, and sample sizes. The conclusions must be drawn appropriately based on the data presented.*

*Reviewer #3: Yes*

 We thank the reviewer for noting this.

*3. Has the statistical analysis been performed appropriately and rigorously?*

*Reviewer* *#3: I Don't Know*

*4. Have the authors made all data underlying the findings in their manuscript fully available?

The* [*PLOS Data policy*](http://www.plosone.org/static/policies.action#sharing) *requires authors to make all data underlying the findings described in their manuscript fully available without restriction, with rare exception (please refer to the Data Availability Statement in the manuscript PDF file). The data should be provided as part of the manuscript or its supporting information, or deposited to a public repository. For example, in addition to summary statistics, the data points behind means, medians and variance measures should be available. If there are restrictions on publicly sharing data—e.g. participant privacy or use of data from a third party—those must be specified.*

*Reviewer* #3: Yes

 We thank the reviewer for noting this.

*5. Is the manuscript presented in an intelligible fashion and written in standard English?

PLOS ONE does not copyedit accepted manuscripts, so the language in submitted articles must be clear, correct, and unambiguous. Any typographical or grammatical errors should be corrected at revision, so please note any specific errors here.*

*Reviewer #3: Yes*

 We thank the reviewer for noting this.

*6. Review Comments to the Author

Please use the space provided to explain your answers to the questions above. You may also include additional comments for the author, including concerns about dual publication, research ethics, or publication ethics. (Please upload your review as an attachment if it exceeds 20,000 characters)*

*Reviewer #3: thank you for this much improved version i have two very minor comments:

Writing style, please ensure this is checked for example on page 35 the authors write "the studies reviewed in (29)" you need to refer to the name of the author here and not only the reference number or write in a previous review (29) ....

i don't feel too strongly but i would question the need for the following sentence to be included: "These findings are important because females have a higher chance of
being an APOE 4 genotype carrier which when combined with cerebrovascular disease (also
higher among women in later age) (109), is predictive of higher likelihood of Alzheimer’s
Disease (AD) in women (110, 111) making spousal males more likely to become caregiver."*

We thank the reviewer for their comments, and we agree we needed an additional round of revisions to address the language, we added Frias and colleagues to the citation for the study previously identified only as 29. In addition, we have deleted the sentence suggested in the discussion.

*While revising your submission, please upload your figure files to the Preflight Analysis and Conversion Engine (PACE) digital diagnostic tool,* [*https://pacev2.apexcovantage.com/*](https://pacev2.apexcovantage.com/)*. PACE helps ensure that figures meet PLOS requirements. To use PACE, you must first register as a user. Registration is free. Then, login and navigate to the UPLOAD tab, where you will find detailed instructions on how to use the tool. If you encounter any issues or have any questions when using PACE, please email P*LOS at [figures@plos.org](mailto:figures@plos.org). Please note that Supporting Information files do not need this step.

 Shoshanna can you check this and update this section?
