# Supplementary material for: Digital tools for delivery of dementia education for caregivers of persons with dementia: A systematic review and meta-analysis of impact on caregiver distress and depressive symptoms
Source: PLoS One. 2023 May 17;18(5):e0283600. doi: 10.1371/journal.pone.0283600 (PMC10191337; doi:10.1371/journal.pone.0283600)
Supplement: Attachment — Submitted filename: Response to Reviewers.docx [file pone.0283600.s012.docx]

PONE-D-21-03124R2

Digital Tools for Delivery of Dementia Education for Caregivers of Persons with Dementia: A Systematic Review and Meta-Analysis

PLOS ONE

Dear Dr. Ammal Mokhtar Metwally, Ph.D (MD)
Academic Editor
PLOS ONE

We thank you for the opportunity to revise this manuscript further. We have addressed all reviewer comments below, and we believe the reviewer comments have contributed to an improved iteration of this manuscript.

- A rebuttal letter that responds to each point raised by the academic editor and reviewer(s). You should upload this letter as a separate file labeled 'Response to Reviewers'.
- A marked-up copy of your manuscript that highlights changes made to the original version. You should upload this as a separate file labeled 'Revised Manuscript with Track Changes'.
- An unmarked version of your revised paper without tracked changes. You should upload this as a separate file labeled 'Manuscript'

**Comments to the Author**

1. If the authors have adequately addressed your comments raised in a previous round of review and you feel that this manuscript is now acceptable for publication, you may indicate that here to bypass the “Comments to the Author” section, enter your conflict of interest statement in the “Confidential to Editor” section, and submit your "Accept" recommendation.

Reviewer #3: All comments have been addressed

Reviewer #4: (No Response)

2. Is the manuscript technically sound, and do the data support the conclusions?

The manuscript must describe a technically sound piece of scientific research with data that supports the conclusions. Experiments must have been conducted rigorously, with appropriate controls, replication, and sample sizes. The conclusions must be drawn appropriately based on the data presented.

Reviewer #3: Yes

Reviewer #4: Partly

Author’s response: We believe the sensitivity analysis suggested by a reviewer will help to reassure all regarding how the data support the conclusions.

3. Has the statistical analysis been performed appropriately and rigorously?

Reviewer #3: Yes

Reviewer #4: Yes

4. Have the authors made all data underlying the findings in their manuscript fully available?

The [PLOS Data policy](http://www.plosone.org/static/policies.action#sharing) requires authors to make all data underlying the findings described in their manuscript fully available without restriction, with rare exception (please refer to the Data Availability Statement in the manuscript PDF file). The data should be provided as part of the manuscript or its supporting information, or deposited to a public repository. For example, in addition to summary statistics, the data points behind means, medians and variance measures should be available. If there are restrictions on publicly sharing data—e.g. participant privacy or use of data from a third party—those must be specified.

Reviewer #3: Yes

Reviewer #4: Yes

5. Is the manuscript presented in an intelligible fashion and written in standard English?

PLOS ONE does not copyedit accepted manuscripts, so the language in submitted articles must be clear, correct, and unambiguous. Any typographical or grammatical errors should be corrected at revision, so please note any specific errors here.

Reviewer #3: Yes

Reviewer #4: Yes

6. Review Comments to the Author

Please use the space provided to explain your answers to the questions above. You may also include additional comments for the author, including concerns about dual publication, research ethics, or publication ethics. (Please upload your review as an attachment if it exceeds 20,000 characters)

Reviewer #3: thank you for this article i am happy that all queries have been responded to and address. a good article which is interesting to read.

Author’s response: We thank reviewer 3 for noting that the revisions addressed the previous reviewers comments.

Reviewer #4: 1. This manuscript has its clear good purposes for caregivers, i.e., analyzing impacts of technology-based dementia education on several life aspects (e.g., depression, distress, self-efficacy) of caregivers through meta-analysis. Regarding the present title, it could be a little vague for potential readers to quickly grasp what the main “outcomes” of interest (e.g., depression) that this paper would like to focus.

Author’s response: This was a helpful suggestion; we had revised the title a few rounds of revision ago to focus on specific outcomes. Digital tools for delivery of dementia education for caregivers of persons with dementia: A systematic review and meta-analysis of impact on caregiver distress and depressive symptoms

2. Introduction: Based on existing literature, it is evidently that digital modes of dementia have already been proven effective in terms of some aspects. And it is understandable that this study wanted to summarize relevant studies to serve as a step toward future education programs. However, walking through the Introduction section, it is still unclear: (1) in this area, what were the exact points which the past research has not been solved/answered; (2) so for this “pooled” study, what “expected” findings/knowledge different from those “single” studies in the past would bring into this area; and (3) what special view(s) that this review can give but past studies couldn’t? I think these should be said in a more systematic and deeper way in Introduction.

Author’s response: We agree with this reviewer – we needed to be clearer. The revised manuscript points out the links more clearly between the literature review, the main aims, and the objectives.

3. Study objectives and search strategy: I would like to suggest the three main questions this review addressed needing to have a strong link back to the Introduction. In other words, it would be better that each of them should have a background to let potential readers clearly understand why this question would be asked?

Author’s response: We hope the revised introduction addresses this concern.

4. Study inclusion and exclusion criteria: dementia has different sub-types, so caregivers of persons with different sub-types may have different burdens. Because of this, even the same intervention may lead to different effectiveness, thus could be a reason biasing the “pooled” result. It is unclear that how the authors address this issue in searching, including, and excluding studies.

Author’s response: This is an excellent point and we have added the fact that the literature review focussed on dementia due to any etiology as a limitation for our findings.

5. Meta-analysis: I would encourage to do some sensitivity analysis to test the stability and robustness of the “pooled” result, for example, study quality, etc.

Author’s response: This is an excellent point, and we performed the below sensitivity analysis. We briefly described the sensitivity analysis in the revised results.

Risk of bias improved after removal of Gallagher-Thompson, Glueckauf, Finkel, Kwok and Boots – for sensitivity analysis.

Risk of bias assessment for RCT studies included in meta-analysis (before removal of studies)

|  | Unknown Risk of Bias | Low Risk of Bias | High Risk of Bias |
| --- | --- | --- | --- |
| Random Sequence Generation | 57 | 36 | 7 |
| Allocation Concealment | 71 | 22 | 7 |
| Blinding of Participants | 65 | 28 | 7 |
| Blinding of Outcome Assessment | 93 | 7 | 0 |
| Incomplete Outcome Data | 0 | 64 | 36 |
| Selective Reporting | 0 | 93 | 7 |

Risk of bias assessment for RCT studies included in meta-analysis (after removal of studies)

|  | Unknown Risk of Bias | Low Risk of Bias | High Risk of Bias |
| --- | --- | --- | --- |
| Random Sequence Generation | 66 | 34 | 0 |
| Allocation Concealment | 77 | 23 | 0 |
| Blinding of Participants | 77 | 23 | 0 |
| Blinding of Outcome Assessment | 100 | 0 | 0 |
| Incomplete Outcome Data | 0 | 100 | 0 |
| Selective Reporting | 0 | 100 | 0 |

New Figure; for sensitivity analysis

Risk of bias assessment for RCT studies included in meta-analysis

|  | Unknown Risk of Bias | Low Risk of Bias | High Risk of Bias |
| --- | --- | --- | --- |
| Random Sequence Generation | 66 | 34 | 0 |
| Allocation Concealment | 77 | 23 | 0 |
| Blinding of Participants | 77 | 23 | 0 |
| Blinding of Outcome Assessment | 100 | 0 | 0 |
| Incomplete Outcome Data | 0 | 100 | 0 |
| Selective Reporting | 0 | 100 | 0 |

*Figure 1.3* Depression assessed with: Center for Epidemiological Studies Depression Scale (CES-D)

*Note:* In the above figure, Mean is the study mean for each group, SD is the standard deviation for each group, Total is the total number (*N*) in each group, Weight is the influence of studies on overall meta-analysis, Std. Mean Difference is the overall effect, Heterogeneity (I^2^) = 0%, *p* value indicates level of statistical significance.

*Figure 1.5* Behavioral problems assessed with: Revised Memory and Behavior Problem Checklist (RMBPC)

*Note:* In the above figure, Mean is the study mean for each group, SD is the standard deviation for each group, Total is the total number (*N*) in each group, Weight is the influence of studies on overall meta-analysis, Std. Mean Difference is the overall effect, Heterogeneity (I^2^) = 88%, *p* value indicates level of statistical significance.

*Figure 1.6* Caregiving Self-Efficacy assessed with: Caregiving Self-Efficacy Scale (CSES)

*Note:* In the above figure, Mean is the study mean for each group, SD is the standard deviation for each group, Total is the total number (*N*) in each group, Weight is the influence of studies on overall meta-analysis, Std. Mean Difference is the overall effect, Heterogeneity (I^2^) = 32%, *p* value indicates level of statistical significance.

6. Discussion: it would be better if this manuscript can strengthen the discussion regarding future dementia and education policy implications about this topic.

Author’s response: We have added implications of these data in the revised discussion.

7. PLOS authors have the option to publish the peer review history of their article ([what does this mean?](https://journals.plos.org/plosone/s/editorial-and-peer-review-process#loc-peer-review-history)). If published, this will include your full peer review and any attached files.

If you choose “no”, your identity will remain anonymous but your review may still be made public.


**Do you want your identity to be public for this peer review?** For information about this choice, including consent withdrawal, please see our [Privacy Policy](https://www.plos.org/privacy-policy).

Reviewer #3: No

Reviewer #4: No

While revising your submission, please upload your figure files to the Preflight Analysis and Conversion Engine (PACE) digital diagnostic tool, <https://pacev2.apexcovantage.com/>. PACE helps ensure that figures meet PLOS requirements. To use PACE, you must first register as a user. Registration is free. Then, login and navigate to the UPLOAD tab, where you will find detailed instructions on how to use the tool. If you encounter any issues or have any questions when using PACE, please email PLOS at [figures@plos.org](mailto:figures@plos.org). Please note that Supporting Information files do not need this step.
